# The Protective Role of Hydrogen Sulfide and Its Impact on Gene Expression Profiling in Rat Model of COPD

**DOI:** 10.1155/2022/9407927

**Published:** 2022-03-18

**Authors:** Yanjing He, Yun Sun, Chengcheng Liao, Fan Lin, Zhengyuan Xia, Yongfen Qi, Yahong Chen

**Affiliations:** ^1^Department of Pulmonary and Critical Care Medicine, Peking University Third Hospital, Beijing, China; ^2^Department of Anesthesiology, The University of Hong Kong, Hong Kong, China; ^3^State Key Laboratory of Pharmaceutical Biotechnology, Department of Medicine, The University of Hong Kong, Hong Kong, China; ^4^Department of Anesthesiology, Affiliated Hospital of Guangdong Medical University, Zhanjiang, China; ^5^Key Laboratory of Molecular Cardiovascular Science, Ministry of Education, Beijing, China

## Abstract

Chronic obstructive pulmonary disease (COPD) is a leading cause of death worldwide, which is usually caused by exposure to noxious particles or gases. Hydrogen sulfide (H_2_S), as an endogenous gasotransmitter, is involved in the pathogenesis of COPD, but its role in COPD is little known. To investigate the role of H_2_S in COPD, a rat model of COPD was established by cigarette smoking (CS) and intratracheal instillation of lipopolysaccharide (LPS). Rats were randomly divided into 4 groups: control, CS + LPS, CS + LPS + sodium hydrosulfide (NaHS, H_2_S donor), and CS + LPS + propargylglycine (PPG, inhibitor of cystathionine-*γ*-lyase, and CTH). Lung function *in vivo*, histology analysis of lung sections, malondialdehyde (MDA) concentration, CTH protein, total superoxide dismutase (T-SOD), and catalase (CAT) activity in lung tissues were assessed. Gene expression profiling of lung was assessed by microarray analysis. The results showed that rats in the CS + LPS group had lower body weight and lung function but higher lung pathological scores, MDA concentration, CTH protein, T-SOD, and CAT activity compared with the control. Compared with CS + LPS group, NaHS treatment decreased lung pathological scores and MDA concentration, while PPG treatment decreased body weight of rats and T-SOD activity, and no significant differences were detected in pathological scores by PPG treatment. Microarray analysis identified multiple differentially expressed genes, and some genes regulated by H_2_S were involved in oxidative stress, apoptosis, and inflammation pathways. It indicates that H_2_S may play a protective role in COPD via antioxidative stress and antiapoptosis pathway.

## 1. Introduction

Chronic obstructive pulmonary disease (COPD) is a preventable and chronic airway disease, which is characterized by airflow limitation that is not fully reversible and persistent respiratory symptoms, and it is usually caused by exposure to noxious particles or gases. Currently, COPD is the third leading cause of death worldwide and causes great burden to the society [1, 2]. Nowadays, it is well-known that cigarette smoking is the commonest risk factor for COPD; however, not all smokers get COPD in their lifetime [3]. In contrast, nonsmokers may develop COPD [4], and it indicates that genetic susceptibility may exist in this complex disease. Other risk factors, such as infections [5], genetic factors (alpha-1 antitrypsin deficiency) [6], and age [7], also influence the development of COPD, while the mechanisms of those risk factors leading to the development of COPD are poorly understood. Oxidative stress [8], apoptosis [9], inflammation [10], and the imbalance of protease and antiprotease [11], all contribute to COPD, and it seems promising to develop an effective antioxidative stress or antiapoptosis intervention as therapies for COPD.

Hydrogen sulfide (H_2_S) was considered as a toxic gas once time, it was discovered as the third endogenous gasotransmitter after nitric oxide (NO) and carbon monoxide (CO) in recent decades [12], and it can be catalyzed and generated in mammalian cells by three enzymes, namely cystathionine-*γ*-lyase (CTH), cystathionine-*β*-synthase (CBS), and 3-mercaptopyruvate sulphurtransferase (3-MST) [13, 14], and the 3-MST is identified in brain and blood vessel wall, this enzyme cooperates with cysteine aminotransferase to produce H_2_S [15]. Accumulated evidence shows that H_2_S is implicated in respiratory diseases, such as COPD, asthma, pulmonary fibrosis, and pulmonary arterial hypertension [16–21]. As an endogenous gasotransmitter, H_2_S is involved in physiological and pathological processes, such as tracheal tone [22], pulmonary fibrosis [20], oxidative stress [23], apoptosis, and inflammation [24]. Our previous study showed that serum H_2_S level was higher in patients with stable COPD than that in healthy control subjects or patients with acute exacerbation of COPD (5), and other study reported that H_2_S protected against tobacco smoke-induced emphysema in mice [25]. However, the underling molecular mechanism of H_2_S in regulating COPD is largely unknown.

In the present study, we established a rat model of COPD and applied microarray analysis to explore potential candidate genes mediated by H_2_S in this model. The potential differentially expressed (DE) genes were screened among the control group, rat model of COPD, and rat model treated with sodium hydrosulfide (NaHS) or propargylglycine (PPG). We hypothesized that H_2_S may play a protective role in COPD by antioxidative stress and antiapoptosis pathways. We further applied Gene Ontology (GO) and Kyoto Encyclopedia of Genes and Genomes (KEGG) enrichment analysis to investigate the signaling pathways which the genes were involved in.

## 2. Materials and Methods

### 2.1. Rat Model of COPD

Animal care and experimental protocols were in compliance with the PR China Animal Management Rule (documentation 55, 2001, Ministry of Health of PR China).

Male Sprague–Dawley (SD) rats (210–250 g) were supplied by the Animal Center, Health Science Center, Peking University, and housed on a 12 h light/dark cycle in a temperature-controlled room (25 ± 2°C) with free access to water and food. The rats were randomly divided into four groups: control, cigarette smoking (CS) + lipopolysaccharide (LPS, Sigma), CS + LPS + NaHS (Sigma), and CS + LPS + PPG (Sigma) groups. The rat model of COPD was established with minor modification as previously described [22, 26]. Briefly, rats were exposed to cigarette smoking in a dynamic smoke box (Tianjin Hope Corp., Tianjin, China) on days 1-4, 6-18, and 20-30, respectively, 2 times/day, 1.5 h/time. In addition, LPS solution (500 *μ*g/ml, 200 *μ*l/animal/time) was instilled into exposed trachea with 1 ml syringe on days 5 and 19. Freshly prepared NaHS (14 *μ*mol/kg) or PPG (37.5 mg/kg) was injected intraperitoneally 2 h before CS or LPS exposure in NaHS or PPG treatment group, respectively. The control group underwent an identical schedule but received air or saline instead. Rats were anesthetized by intraperitoneal injection of 20% (w/v) urethane (5 ml/kg) 24 h later after the last CS exposure and processed to lung function test. The rats were sacrificed by exsanguination from abdominal artery.

### 2.2. Lung Function Test and Histological Analysis

Lung function was tested as previously described [18]. Peak expiratory flow (PEF), peak inspiratory flow (PIF), intrapressure (IP), and maximum rising slope of IP (IP slope) were analyzed using Chart 4.1 software (AD Instruments, Australia).

Histological analysis of rat lung and pathological scores was performed as previously described [22]. Briefly, the left lung tissue was fixed in 4% paraformaldehyde solution and embedded in paraffin. The lung tissue slices (5 *μ*m) were stained with hematoxylin and eosin (H&E), periodic acid-schiff (PAS), and Masson trichrome separately. The pathological scores were evaluated according to the following nine parameters with mild modification, namely constriction or occlusion of the small airway lumen, abscission or ulceration, squamous cell metaplasia, goblet cell proliferation of epithelium, inflammatory cell infiltration, pigment deposits, proliferation of fibrous tissue, smooth muscle hypertrophy in the airway wall, and emphysema of lung tissue. Three membranous bronchioles of each rat were selected for evaluation at random. Score for each parameter was ranged from 0 (normal) to 3 (abnormal). A total pathological score is calculated by summing the scores to all nine parameters in each rat. Scoring was performed in a blinded test.

### 2.3. MDA Concentration, Total SOD Activity, and CAT Activity

Rat lung tissues stored in -80°C fridge were homogenized in cold sterile saline on ice. Malondialdehyde (MDA) concentration, total superoxide dismutase (T-SOD), and catalase (CAT) activity were measured by spectrophotometry according to manufacturer's instructions (Jiancheng Bioengineering Institute, Nanjing, China) as previously described [27]. The MDA concentration was expressed as nmol.mg^−1^ protein. The T-SOD and CAT activity were expressed as units.mg^−1^ protein.

### 2.4. Western Blotting Analysis

The protein extraction of lung tissue was boiled with gel-loading buffer for 10 min at 100°C, resolved on 12% SDS-PAGE, and transferred to nitrocellulose membranes. The membranes were probed with CTH antibody (1 : 2000, Abnova, Taiwan) or *β*-actin antibody (Santa Cruz Biotechnology) and visualized with enhanced chemiluminescence (Applygen Technologies; Beijing). The protein levels were quantified by Image J software and normalized to *β*-actin as an internal control.

### 2.5. RNA Extraction and Microarray Analysis

Total RNA was extracted from the left upper lobe of rat lungs using Trizol reagent (Invitrogen, Carlsbad, CA) according to the manufacturer's instructions. The purity and concentration of RNA was detected using NanoDrop 2000 spectrophotometer. The quality of RNA was assessed using Agilent 2100 Bioanalyzer, and samples with a RIN (RNA integrity number) of >7.0 were used in this study. cRNA was hybridized against Agilent G4853A GeneChips. Microarray experiments were performed following manufacturer's instructions. The raw data was analyzed using GeneSpring version 12.0 software. The screened differentially expressed DE genes were processed to cluster analysis, GO, and KEGG enrichment analysis using Funnet (http://www.funnet.info).

### 2.6. Statistical Analysis

Data are expressed as mean ± standard deviation (SD) or mean ± standard error of mean (SEM). Statistical significance in pathological scores among groups is tested with Kruskal-Wallis test followed by Dunn's post test. Other comparisons among groups are tested with one-way analysis of variance followed by Turkey test. Unpaired *t*-test is used between the two groups (GraphPad prism version 5). To derive DE genes, the fold change ≧2 is considered as upregulation or downregulation. A value of *P* < 0.05 is considered statistically significant.

## 3. Results

### 3.1. Body Weight and Lung Function

The COPD rat model was established by continuous cigarette smoking and intratracheal instillation of lipopolysaccharide (LPS) for 2 times. The body weight of each rat was monitored each day before the administration of NaHS or PPG.

Before experiment, there was no significant difference in body weight of rats among the groups. By the end of the experiment, the body weight in the CS + LPS group was decreased significantly compared with the control. Compared with the CS + LPS group, there was no significance in body weight of rats by NaHS treatment, but PPG treatment decreased body weight markedly (Table 1).

For lung function, The peak expiratory flow (PEF) was significantly lower in the CS + LPS group than that in the control group, whereas intrapressure (IP) was 66% higher (all *P* < 0.01) (Table 2). No significant differences were detected in lung function after NaHS or PPG treatment compared with the CS + LPS group (Table 2).

Data are expressed as mean ± SD, *n* = 7/group; ^∗^*P* < 0.05 vs. control, ^∗∗∗^*P* < 0.001 vs. CS + LPS group.

PIF, peak inspiratory flow; PEF, peak expiratory flow; IP, intrapressure; IP slope, maximum rising slope of IP. Data are expressed as mean ± SD, *n* = 7/group, ^∗∗^*P* < 0.01, and ^∗∗∗^*P* < 0.001 vs. control.

### 3.2. NaHS Treatment Alleviated Lung Injury in Rats Exposed to CS Combined with LPS

**Figure 1:**
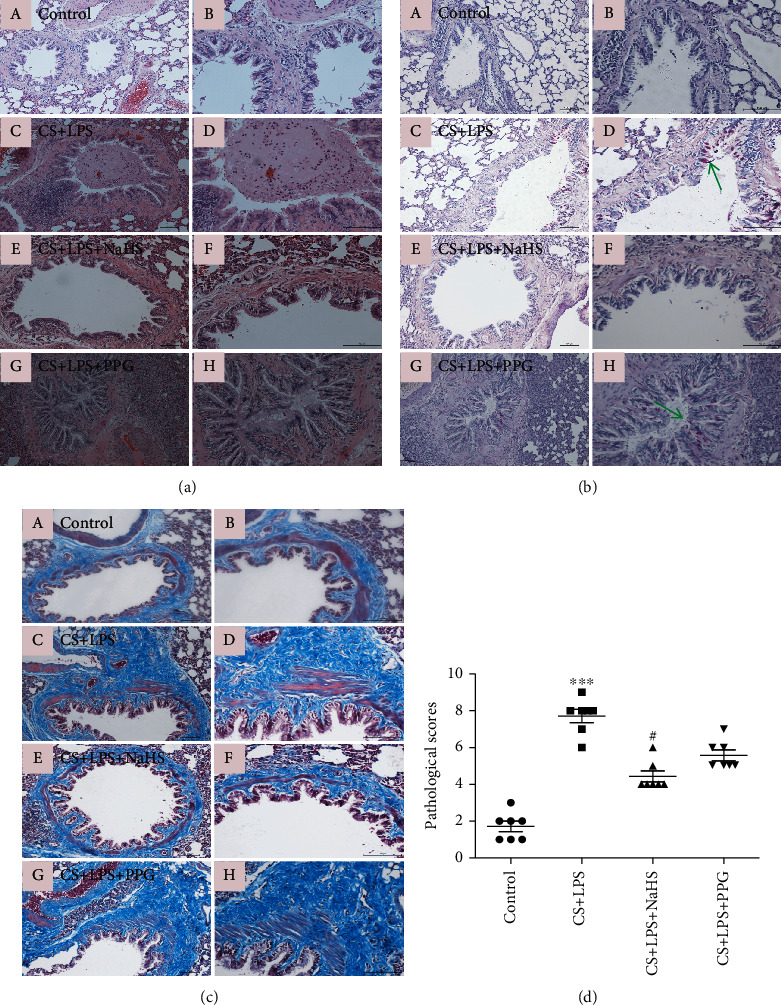
Histological analysis of lung tissue section. (a) H&E staining. (b) PAS staining, arrows indicate positive staining. (c) Masson trichrome staining. (A) and (B): control group; (C) and (D): CS + LPS group; (E) and (F): CS + LPS + NaHS group; (G), (H): CS + LPS + PPG group. (A), (C), (E), and (G) (magnification ×100); (B), (D), (F), and (H) (magnification ×200). (d) Pathological scores of rat lung tissues. Data are expressed as mean ± SEM (*n* = 7/group), ^∗∗∗^*P* < 0.001 vs. control; ^#^*P* < 0.05 vs. CS + LPS group.

In CS + LPS group, represented COPD rat model, the lung sections by histological analysis showed the following pathological features: airway mucus secretion and inflammatory cell obstruction, necrosis and erosion of bronchial epithelium, metaplasia of airway goblet cells, airway inflammatory cell infiltration, proliferation of fibrous tissues in airway wall, and emphysema, and each features was shown in different degrees. Chronic bronchitis was the main feature of this model. The lung pathological injury scores in the CS + LPS group were increased significantly compared with that in the control group (*P* < 0.01) (Figures 1(a), 1(b), 1(c), and 1(d)), whereas the scores were decreased by NaHS treatment (*P* < 0.01). No significant difference was detected in pathological scores between CS + LPS and CS + LPS + PPG group. The raw histological images were provided in supplementary files (SF1).

### 3.3. Oxidant/Antioxidant Stress Imbalance

**Figure 2:**
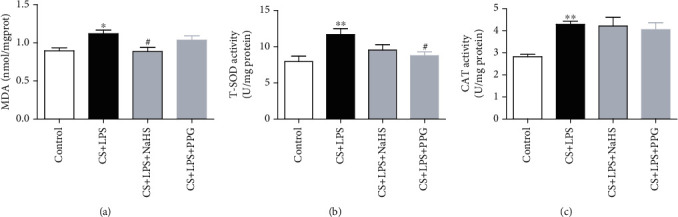
MDA concentration and T-SOD and CAT activities in rat lung tissues. (a) MDA concentration; (b) T-SOD activity; (c) CAT activity. Data are expressed as mean ± SEM (*n* = 7/group), ^∗^*P* < 0.05, ^∗∗^*P* < 0.01 vs. control, and ^#^*P* < 0.05 vs. CS + LPS group.

MDA, SOD, and CAT activities were selected to act as markers of oxidative stress. Compared with the control, in the CS + LPS group, the MDA concentration in lung tissues was increased by 24% (*P* < 0.05) (Figure 2(a)); compared with the CS + LPS group, NaHS treatment decreased MDA concentration by 21% (*P* < 0.05), whereas there is no significant difference for MDA concentration after the blockade of endogenous CTH with PPG; compared with the control group, the T-SOD and CAT activity in the CS + LPS group were increased by 47% and 52% (*P* < 0.01), respectively; However, compared with the CS + LPS group, after the administration of NaHS or PPG, there is no significantly difference for CAT activity among the groups, but the T-SOD activity was decreased by 33% in the CS + LPS + PPG group compared with the CS + LPS group (*P* < 0.05) (Figures 2(b) and 2(c)).

### 3.4. Chronic Exposure to CS Combined with LPS Increased CTH Protein Expression Level

**Figure 3:**
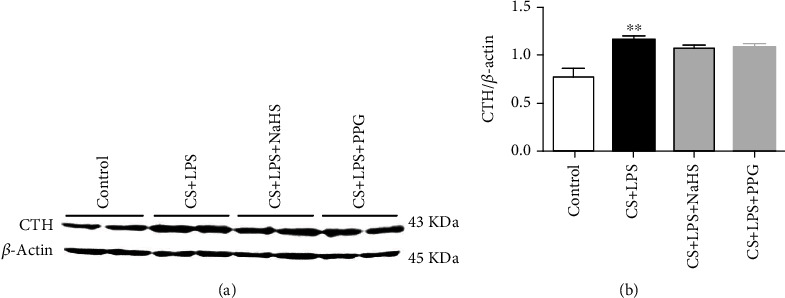
CTH protein in rat lung tissues. (a) Representative band of western blotting. *β*-Actin was reference control. (b) Quantification of CTH protein level by Image J software. Data are expressed as mean ± SEM (*n* = 4/group), ^∗∗^*P* < 0.01 vs. control.

CTH protein expression level in lung tissue was increased in the CS + LPS group compared with the control group (*P* < 0.01) (Figure 3); however, after the administration of NaHS or PPG, there was no statistical significance for CTH protein level compared with CS + LPS group.

### 3.5. DE Genes Screened by Microarray Analysis

There were total 30507 genes on gene chips, of which 20726 genes were detected and 9781 genes were not detected. Genes with fold change (FC) ≥ 2 and *P* <0.05 were accepted as DE genes. The gene expression data was deposited in Gene Expression Omnibus (GEO), and the GEO accession number is GSE184693 (https://www.ncbi.nlm.nih.gov/geo/info/linking.html). Comparing the CS + LPS group with control, 341 DE genes were identified, of these 341 genes, 108 genes were downregulated, and 233 genes upregulated; comparing the CS + LPS + NaHS or CS + LPS + PPG group with the control group, 241 and 448 DE genes were identified, respectively; comparing the CS + LPS + NaHS group with the CS + LPS group, 191 DE genes were identified, of these 191 genes, 40 genes were downregulated, and 151 genes upregulated; comparing the CS + LPS + PPG group with the CS + LPS group, 217 DE genes were identified, of these 217 genes, 56 genes were downregulated, and 161 genes upregulated (Table 3).

### 3.6. Cluster Analysis

**Figure 4:**
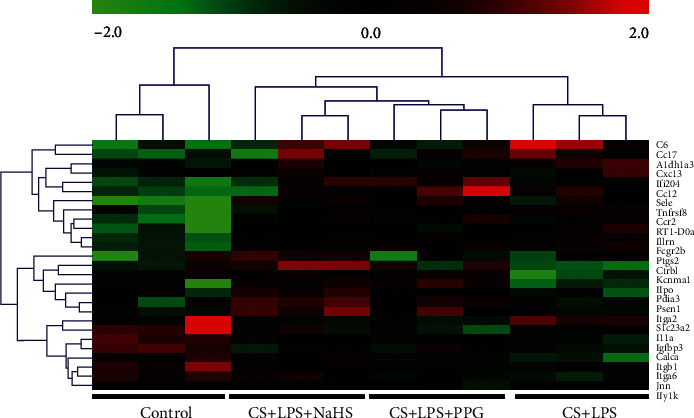
Heat map of unsupervised hierarchical cluster analysis of representative genes. Data from individual subjects and individual genes are shown in columns and rows separately. The color represents the intensity of the signal (*n* = 3/group). Green bars represent low expression levels, red bars represent high expression levels, and black bars represent no change relative to normalized median gene expression values.

The genes were processed to unsupervised hierarchical cluster analysis. Generally, subjects with similar characteristics will cluster together into the same cluster, and the DE genes with similar expression patterns will cluster together, and we found the subjects in the same group were clustered together. Thereafter, all the DE genes were annotated by GO and KEGG enrichment analysis (in the following sections), 27 DE genes involved in apoptosis, oxidative stress, and inflammation, and engulfment pathways were selected to cluster again with unsupervised hierarchical cluster analysis; it showed that subjects in the same group clustered together, the gene expression profiling between the CS + LPS and control group has big difference, and the gene expression profiling in theCS + LPS + NaHSgroup was close to the control group (Figure 4).

### 3.7. GO Enrichment Analysis

The GO enrichment analysis includes biological process category, molecular function category, and cellular component category. All the DE genes were annotated by GO enrichment analysis.

**Figure 5:**
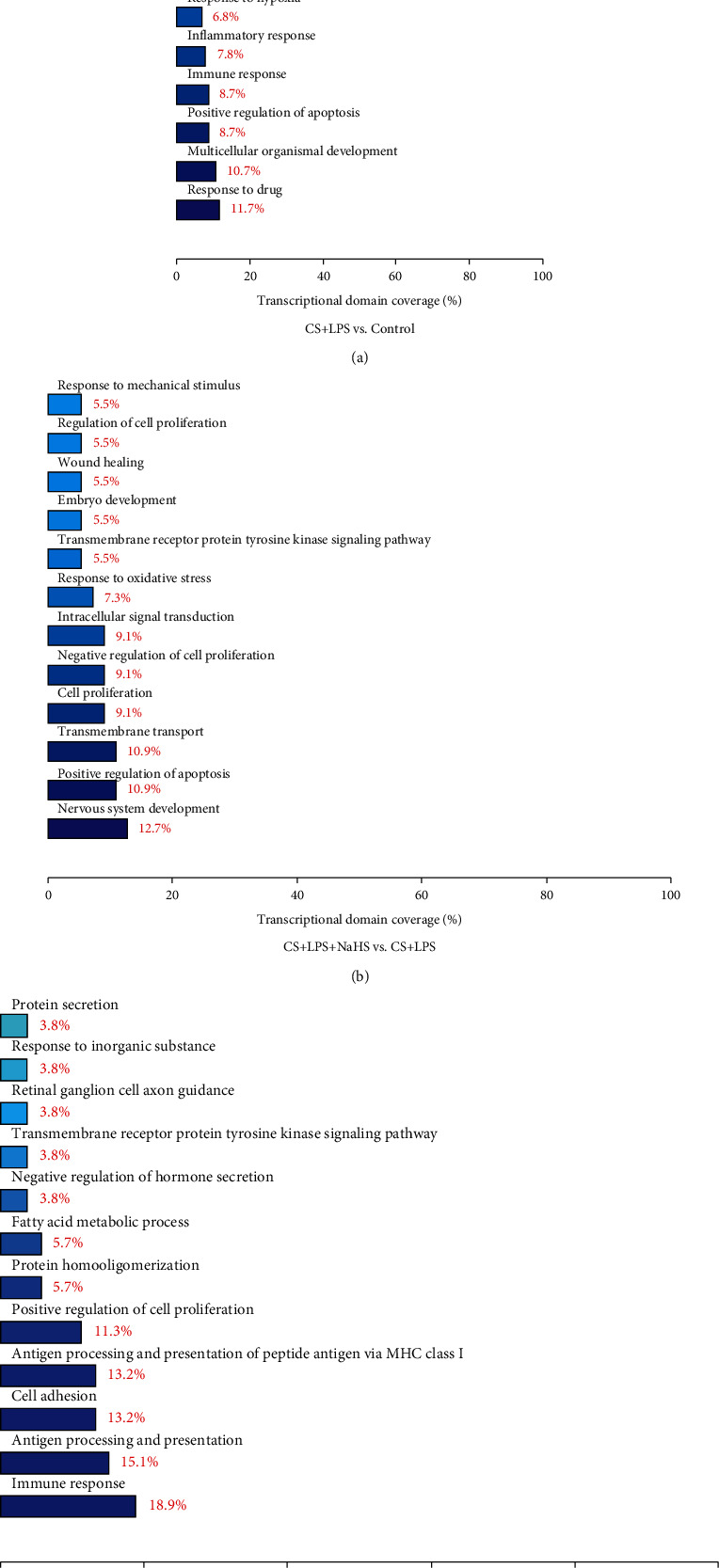
DE genes annotated by the considered GO biological process category. The DE genes between CS + LPS and control group (a), between CS + LPS + NaHS and CS + LPS group (b), and between CS + LPS + PPG and CS + LPS group (c), respectively. (*n* = 3/group).

Between CS + LPS group and control group, 163 DE genes were annotated in GO biological processes. According to the number of DE genes annotated by the considered GO biological process category, the top 5 signaling pathways were shown as follows: response to drug (12 genes), multicellular organismal development (11 genes), positive regulation of apoptosis (9 genes), immune response (9 genes), and inflammatory response (8 genes) (Figure 5(a)). The DE genes involved in positive regulation of apoptosis pathway are *Itga6* (integrin, alpha6), *C6* (complement component 6), *Igfbp3* (insulin-like growth factor binding protein 3), *Itgb1* (integrin, beta1), *Jun* (Jun oncogene), *Tnfrsf8* (tumor necrosis factor receptor superfamily, member 8), *Aldh1a3* (aldehyde dehydrogenase 1 family, member A3), *Ifi204* (interferon activated gene 204), and *Kcnma1* (potassium large conductance calcium-activated channel, subfamily M, alpha member1) (Table 4).

Between CS + LPS + NaHS group and CS + LPS group, 92 DE genes were annotated in GO biological process, and the top 5 signaling pathways were shown as follows: nervous system development (7 genes), positive regulation of apoptosis (6 genes), transmembrane transport (6 genes), cell proliferation (5 genes), and negative regulation of cell proliferation (5 genes) (Figure 5(b)). The DE genes involved in positive regulation of apoptosis pathway are *Itgb1*, *Kcnma1*, *Psen1* (presenilin 1), *Pdia3* (protein disulfide isomerase family A member 3), *Ptgs2* (prostaglandin-endoperoxide synthase 2), and *Ctrb1* (chymotrypsinogen B1) (Table 4). Notably, *Itgb1* and *Kcnma1* were downregulated in the CL + LPS group compared with the control group but upregulated in the NaHS-treated group compared with the CL + LPS group. Between CS + LPS + NaHS group and CS + LPS group, there were 4 genes involved in response to oxidative stress pathway, namely *Psen1*, *Ptgs2* (prostaglandin endoperoxide synthase 2), *Mpo* (myeloperoxidase), and *Slc23a2* (solute carrier family 23 member 2), the former three genes were upregulated in the NaHS treatment group compared with the CS + LPS group, and the latter one was downregulated. The gene *Ppargc1a* (peroxisome proliferator-activated receptor gamma, coactivator 1 alpha) is involved in the regulation of cell death, positive regulation of gluconeogenesis, positive regulation of fatty acid oxidation, negative regulation of neuron apoptosis, and neuron death, and it was upregulated in the NaHS treatment group compared with the CS + LPS group.

Between CS + LPS + PPG group and CS + LPS group, 98 DE genes were annotated in GO biological process, and the top 5 signaling pathways were shown as follows: immune response (10 genes), antigen processing and presentation (8 genes), cell adhesion (7 genes), antigen processing and presentation of peptide antigen via MHC class I (7 genes), and positive regulation of cell proliferation (6 genes) (Figure 5(c)).

The detailed DE genes annotated by considered GO biological process category, GO molecular function category, and cellular component category were in supplementary data online (File S2).

### 3.8. KEGG Enrichment Analysis

All the DE genes were also annotated by the considered KEGG category.

**Figure 6:**
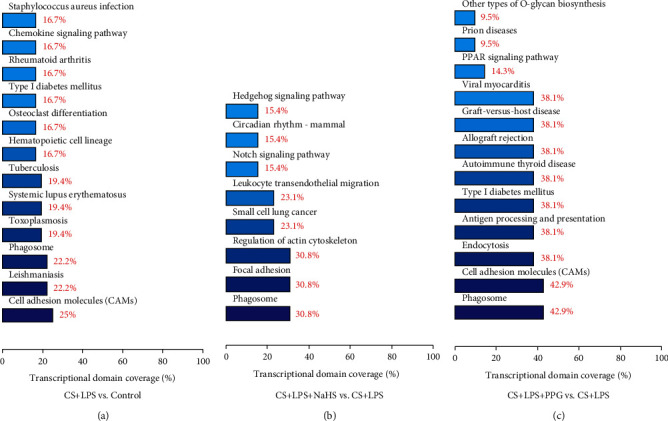
DE genes annotated by the considered KEGG category. The DE genes between CS + LPS and control group (a), between CS + LPS + NaHS and CS + LPS group (b), and between CS + LPS + PPG and CS + LPS group (c), respectively. (*n* = 3/group).

Between CS + LPS group and control group, 85 DE genes were annotated by the considered KEGG category. According to the number of DE genes annotated by the considered KEGG category, the top 5 signaling pathways were shown as follows: cell adhesion molecules (9 genes), leishmaniasis (8 genes), phagosome (8 genes), toxoplasmosis (7 genes), and systemic lupus erythematosus (7 genes) (Figure 6(a)).

Between CS + LPS + NaHS group and CS + LPS group, 39 DE genes were annotated by the considered KEGG category, and the top 5 signaling pathways were shown as follows: phagosome (4 genes), focal adhesion (4 genes), regulation of actin cytoskeleton (4 genes), small cell lung cancer (3 genes), and leukocyte transendothelial migration (3 genes) (Figure 6(b)).

Between CS + LPS + PPG group and CS + LPS group, 52 DE genes were annotated by the considered KEGG category, and the top 5 signaling pathways were shown as follows: phagosome (9 genes), cell adhesion molecules (9 genes), endocytosis (8 genes), antigen processing and presentation (8 genes), and type 1 diabetes mellitus (8 genes) (Figure 6(c)).

The detailed DE genes annotated by considered KEGG enrichment analysis were in supplementary data online (File S3).

## 4. Discussion

In the present study, in order to shorten the induction of COPD rat model, we established the model by exposure to CS and LPS, and histological analysis of lung has shown some pathological features of COPD, such as airway mucus and inflammatory cells obstruction, airway inflammatory cell infiltration, and proliferation of fibrous connective tissues in airway wall, and this model showed the main features of chronic bronchitis. Our findings showed that the pathological scores in the COPD rat model and the lipid peroxidation product MDA were increased significantly compared with the control and reduced by NaHS treatment. The activities of antioxidant enzymes T-SOD and CAT were also increased significantly in the rat model of COPD, and the PPG treatment reduced the activity of T-SOD and body weight. Furthermore, with microarray analysis, we found lots of DE genes were implicated in multiple biological pathways, such as response to oxidative stress and regulation of apoptosis pathways, and some DE genes, such as *Ppargcla*, *Itgb1*, *Kcnma*, *Ptgs2*, and *Psen1*, may play the role of antioxidative stress and antiapoptosis in the rat model of COPD.

Oxidative stress is one of critical mechanisms in the development of COPD [8]. As a lipid peroxidation product, serum MDA concentration was higher in patients with COPD compared with healthy people [28], consistent with previous study, MDA concentration in lung tissue of COPD rat model was higher compared with the control in our study. SOD enzymes include CuZnSOD, MnSOD (SOD2), and SOD3, SOD3 levels in induced sputum supernatants of COPD patients were higher compared to nonsmokers [29], and in our study, both total SOD and CAT activity were increased in COPD rat model, it may be a compensatory mechanism to counteract the acceleration of oxidative stress. H_2_S, as a gasotranmitter following NO and CO, has its own antioxidant features. H_2_S scavenged peroxynitrite and inhibited peroxynitrite-induced tyrosine nitration [30], it enhanced glutathione production in cells and alleviated cell injury induced by oxidative stress [31], and it also alleviated cigarette smoke extraction or LPS-induced oxidative stress [32, 33]. Our study demonstrated that the NaHS treatment reduced the pathological injury scores and MDA concentration compared to the COPD rat model, and the PPG treatment reduced the activity of T-SOD and body weight of rats. It is suggested that H_2_S acts as an antioxidant to protect against oxidative stress in this COPD rat model. The CAT activity was not changed by NaHS or PPG treatment, and the mechanism needs to be investigated in future study.

COPD is a progressive and irreversible disorder, whichever genes involved in its pathogenesis is incompletely understood. The present study applied microarray analysis to investigate the impact of H_2_S on gene expression profiling in COPD. Our study has found some DE genes between NaHS treatment group and CS + LPS group, such as *Psen1*, *Ptgs2*, *Mpo*, and *Slc23a2*, were involved in oxidative stress pathway, and *Ppargc1a* was upregulated by NaHS treatment. The protein encoded by *Ppargc1a* gene (also known as PGC-1*α* in human) is peroxisome proliferator-activated receptor gamma coactivator 1*α* (PGC-1*α*), as a transcriptional coactivator, it is an important regulator of energy metabolism [34], overexpressed PGC-1*α* alleviates oxidative stress and decreases apoptotic cell death in endothelial cells [34], and upregulation of PGC-1*α* is associated with reduction of oxidative stress and inflammation in diabetic myocardium [35] and attenuation of COPD in mice [36]. PGC-1*α* is essential for the induction of ROS-detoxifying enzymes such as glutathione peroxidase and SOD2 under oxidative stress, PGC-1*α* null mice were sensitive to oxidative stress [37]; PGC-1*α* inhibited mitochondrial oxidative stress by facilitating Nrf2 binding to antioxidant response element promoter site and inducing SOD2 expression in sepsis [38]. It is suggested that the upregulation of PGC-1*α* by H_2_S is one of the mechanisms for antioxidative stress and for the treatment of COPD.

Apoptosis is one kind of regulated cell death [9], many studies have shown that apoptotic cells are increased in the lungs of COPD patients [39], and apoptosis of epithelial and endothelial lung cells led to lung parenchyma destruction and emphysema; animal experiments have also proven that apoptosis is involved in the pathogenesis of COPD [40]. In the present study, 6 differential expressed genes between NaHS treatment group and CS + LPS group were involved in the positive regulation of apoptosis pathway (Table 4), such as *Ptgs2* and *Itgb1*, and these two genes were upregulated by NaHS treatment. *Ptgs2* encodes protein prostaglandin-endoperoxide synthase 2 is also known as cyclooxygenase 2 (COX-2). COX-2 is an enzyme that catalyzes the production of thromboxanes and prostaglandins from arachidonic acid, it is related with tumorigenesis, and the overexpression of COX-2 inhibits apoptosis [41]. In contrast, inhibition of COX-2 promoted TGF-*β*-induced apoptosis and aortic valve calcification [42]. In addition, *Itgb1* encodes protein *β*1 integrin, which is one subunit of integrins. Integrins are membrane receptors that mediate cell adhesion and migration. Cultured pancreatic acinar cells from *β*1 integrin-deficient mice showed an increase of cell apoptosis [43]; *β*1 integrin is important for the survival of vascular smooth muscle cells, the conditional deletion of *Itgb1* in adult mice resulted in vascular smooth muscle cell apoptosis and vascular fibrosis [44]; edothelial *β*1 integrin is indispensable for embryonic liver growth, deletion of *Itgb1* in endothelial cells results in smaller liver size and more apoptotic cells in liver [45]. Our previous study has shown that H_2_S attenuated cigarette smoke-induced apoptosis in rat lung [46], and the upregulation of gene *Ptgs2* and *Itgb1* by H_2_S may contribute to attenuate apoptosis in the lungs of COPD rat model. It needs further validation in vitro in future study.

The novelty of the present study is that we screened many DE genes regulated by H_2_S in COPD rat model, and by bioinformatic analysis, we found that the DE genes were involved in multiple signaling pathways, such as oxidative stress, apoptosis, immune response, and inflammation response pathways, and several genes were upregulated by H_2_S treatment. However, there are several drawbacks in this study. The first drawback is that we did not further verify the interesting DE genes by quantitative real-time polymerase chain reaction (qRT-PCR). Another drawback is that we did not detect markers of apoptosis in lung tissues, while our previous study has demonstrated that H_2_S alleviated cigarette smoke-induced apoptosis *in vivo* [46], and the present work has proven the successful COPD rat model induced by cigarette smoking and LPS and detected the markers of oxidative stress.

Future studies are needed to confirm the DE genes regulated by H_2_S in the development of COPD, such as *Ppargc1a, Ptgs2*, and *Itgb1*. It is indispensable to prove the function of these genes *in vivo* and *in vitro*. As microarray analysis screens thousands of genes and gives us too much information, it is meaningful to explore other interesting genes from the data. We afforded preliminary data of DE genes in this study, and the findings will give insight into future study for the mechanisms of H_2_S in COPD.

## Figures and Tables

**Table 1:** The body weight of rats in each group before and after experiment.

Group	Preexperiment (g)	Postexperiment (g)
Control	246.4 ± 6.4	436.7 ± 34.88
CS + LPS	241.5 ± 15.1	384.4 ± 18.36^∗^
CS + LPS + NaHS	241.9 ± 6.1	394.0 ± 24.1
CS + LPS + PPG	239.4 ± 9.4	305.2 ± 37.7^∗∗∗^

**Table 2:** Lung function.

Group	PIF(L/S)	PEF(L/S)	IP(mmHg)	IPslope(mmHg/s)
Control	3.09 ± 0.19	6.13 ± 0.99	0.90 ± 0.42	91.99 ± 16.26
CS + LPS	2.97 ± 0.12	4.68 ± 0.16^∗∗∗^	1.49 ± 0.16^∗∗^	95.52 ± 8.23
CS + LPS + NaHS	2.96 ± 0.10	4.93 ± 0.13	1.33 ± 0.20	91.07 ± 4.92
CS + LPS + PPG	2.96 ± 0.10	4.80 ± 0.27	1.45 ± 0.18	87.67 ± 3.42

**Table 3:** The number of DE Genes.

Comparison group	Reference group	Upregulated	Downregulated	Total
CS + LPS	Control	233	108	341
CS + LPS + NaHS	Control	127	114	241
CS + LPS + PPG	Control	287	161	448
CS + LPS + NaHS	CS + LPS	151	40	191
CS + LPS + PPG	CS + LPS	161	56	217

^∗^ *n* = 3/group.

**Table 4:** The DE Genes involved in positive regulation of apoptosis or response to oxidative stress.

GO biological process		
Positive regulation of apoptosis		Response to oxidative stress
CS + LPS/control	CS + LPS + NaHS/CS + LPS	CS + LPS + NaHS/CS + LPS
*Itgb1*↓	*Itgb1*↑	*Slc23a2*↓
*Kcnma1*↓	*Kcnma1*↑	*Mpo*↑
*Igfbp3*↓	*Ctrb1*↑	*Psen1*↑
*Itga6*↓	*Pdia3*↑	*Ptgs2*↑
*Jun*↓	*Psen1*↑	_
*Aldh1a3*↑	*Ptgs2*↑	_
*C6*↑	_	_
*Ifi204*↑	_	_
*Tnfrsf8*↑	_	_

“↓”:downregulated; “↑”: upregulated.

## Data Availability

The gene expression data used to support the findings of this study are deposited in the database Gene Expression Omnibus (GEO) (GEO accession number: GSE184693). Other data are included within the article and supplementary information files.

## Supplementary Materials

Supplementary Materials File S1: the raw histological images. File S2: the DE genes annotated by considered GO enrichment analysis between CS + LPS and control group, between CS + LPS + NaHS and CS + LPS group, and between CS + LPS + PPG and CS + LPS group. File S3: the DE genes annotated by considered KEGG enrichment analysis between CS + LPS and control group, between CS + LPS + NaHS and CS + LPS group, and between CS + LPS + PPG and CS + LPS group.
