# Supplementary material for: The Protective Role of Hydrogen Sulfide and Its Impact on Gene Expression Profiling in Rat Model of COPD
Source: Oxid Med Cell Longev. 2022 Mar 18;2022:9407927. doi: 10.1155/2022/9407927 (PMC8956388; doi:10.1155/2022/9407927)
Supplement: Supplementary Materials — File S1: the raw histological images. File S2: the DE genes annotated by considered GO enrichment analysis between CS + LPS and control group, between CS + LPS + NaHS and CS + LPS group, and between CS + LPS + PPG and CS + LPS group. File S3: the DE genes annotated by considered KEGG enrichment analysis between CS + LPS and control group, between CS + LPS + NaHS and CS + LPS group, and between CS + LPS + PPG and CS + LPS group. [file 9407927.f1.docx]

**Supplementary materials:**

File S1:

The raw histological images.

(https://drive.google.com/file/d/1SHixLhyMOg4CusYtlIR8hBG6GKT6b_yW/view?usp=sharing).

File S2:

The DE genes annotated by considered GO enrichment analysis between CS+LPS and Control group, between CS+LPS+NaHS and CS+LPS group, and between CS+LPS+PPG and CS+LPS group.

(<https://drive.google.com/file/d/1dAGhowcWb_WPGoNtx4mjwSqm7dLeC8Q4/view?usp=sharing>).

File S3:

The DE genes annotated by considered KEGG enrichment analysis between CS+LPS and Control group, between CS+LPS+NaHS and CS+LPS group, and between CS+LPS+PPG and CS+LPS group.

**(**https://drive.google.com/file/d/1P77f-zdZH_HEisBeQNtpZyLBnAB_BUBj/view?usp=sharing)
